# Suicide in the COVID-19 Pandemic

**DOI:** 10.1192/j.eurpsy.2022.139

**Published:** 2022-09-01

**Authors:** D. Wasserman

**Affiliations:** Karolinska Institutet, National Centre For Suicide Research And Prevention Of Mental Lll-health, Solna, Sweden

**Keywords:** suicide prevention, Suicidal ideation and behaviours, Covid-19

## Abstract

A literature search using PubMed and Google Scholar identified 64 articles assessing suicidal thoughts, suicide attempts and suicide during the COVID-19 pandemic in comparison with the pre-pandemic period in the six WHO regions of the world. Most studies come from high- and middle-income countries. There is a scarcity of data from South America, and no studies from the African and East-Mediterranean Regions. Compared to trends in previous years, suicide rates remained largely unchanged globally or declined in the early phase of the pandemic. However, increased suicide rates were reported among non-white residents and Afro - American groups in the US, as well as among adolescents in China. Japan and India showed a statistically significant increase in suicide rates after an initial decline. Similarly in Peru, after an initial decline, suicide rates increased among men during the course of the pandemic. This is in line with previous findings in the context of natural disasters and other epidemics where a similar increased suicide trend can be expected in the post-pandemic period in other countries. Among adolescents, there were no significant changes in suicide rates during the period of school closure, but an increase has been observed in the period after coming back to schools. The assessment of suicidal thoughts and attempts during the pandemic was mostly conducted through online cross-sectional surveys and showed significant increases, particularly in females and the young. Suicide can be prevented if evidence-based methods that exist are implemented in a systematic way (Wasserman et al. 2020; https://doi.org/10.1002/wps.20801).

**Disclosure:**

No significant relationships.

